# Adults with Longstanding Strabismus: Psychosocial and Functional Impacts and Reasons behind Surgery Delay

**DOI:** 10.1155/2022/8682675

**Published:** 2022-06-16

**Authors:** Rami Al-Omari, Hisham M Jammal, Yousef Khader, Dema Atoum, Wedad Al-dolat, Moawiah Khatatbeh

**Affiliations:** ^1^Department of Ophthalmology, Yarmouk University, Irbid, Jordan; ^2^Department of Ophthalmology, Jordan University of Science and Technology, Irbid, Jordan; ^3^Department of Public Health, Jordan University of Science and Technology, Irbid, Jordan; ^4^Department of Basic Medical Sciences, Yarmouk University, Irbid, Jordan

## Abstract

**Purpose:**

The aims of the study were to determine the reasons behind surgical correction delay in adult patients with strabismus, reveal motivations for seeking treatment, and study the psychosocial and functional impacts of strabismus on patients using an Arabic version of the Adult Strabismus-20 (AS-20) questionnaire. *Methods and Patients.* This study included 79 patients aged ≥18 years and had strabismus for at least one year prior to surgical correction and 40 controls without known visual defects. After a comprehensive ophthalmic exam during their preoperative visit, a validated questionnaire was administered to patients to collect sociodemographic data, reasons for surgery delay, and motivations for seeking treatment now. A translated version of the AS-20 questionnaire was then presented to patients and controls. Total AS-20 (and its subscales: psychosocial and function) scores were calculated and analyzed. All relationships between sociodemographic characteristics, the onset of deviation, presence of diplopia, type and size of deviation, and the changes in the scores of AS-20 (and its subscales) were investigated.

**Results:**

A total of 79 adult patients with strabismus (cases) and 40 subjects with normal vision (control group) were included in this study. The mean age (SD) was 34.10 (11.5) years for cases (range: 18–61) and 34.20 (11.2) years for controls (range: 18–65) (*p*=0.964). About half (55.7%, *n* = 44) of the patients were males compared to (57.5%, *n* = 23) of the controls. The reasons for strabismus surgery delay reported by the patients were the following: surgery was not offered by an ophthalmologist (35.4%), surgery was offered but declined by the patient due to fear from surgical complications (22.8%), nonaffordability (17.7%), surgery was offered but refused because patients thought they were too old for surgery or patient was not bothered by appearance (15.2%), and patient never sought care (8.9%). The reasons for seeking surgical treatment after this delay were as follows: for cosmetic issues (27.8%), a better understanding of strabismus surgery and its potential complications (20.3%), pressure from family and friends (16.5%), improved economic status (13.9%), relationship/marriage prospects (13.9%), and to improve chances of getting a job (7.6%). When compared to control, patients have significantly lower mean scores of total AS-20 (50.57 vs. 88.01) and its psychosocial (49.59 vs. 87.84) and functional (51.55 vs. 88.19) subscales. AS-20 total score was significantly lower among females and in patients with large deviation size (>25 PD). The psychosocial subscale of AS-20 was significantly lower in females, patients with younger age of onset, and those with large deviation size (>25 PD). Female gender, large deviation size, vertical deviation, and having diplopia correlated significantly with a lower functional score.

**Conclusion:**

Strabismus has a profound psychosocial and functional impact on affected individuals, especially females and patients with large deviation sizes. Many adult patients with strabismus tend to delay surgical correction; most of these delays could be avoided by better public education, increased awareness among health care providers, and changing health insurance policies to cover the costs of strabismus surgery.

## 1. Introduction

Adult strabismus is a common ophthalmic disorder affecting as much as 4% of the adult population [1]. Some patients have a history of childhood-onset strabismus that were not surgically corrected or recurred after correction; others develop strabismus after visual maturation secondary to different pathologies, e.g., cranial nerve palsies, trauma, thyroid eye disease, and vision loss.

Several studies have demonstrated the impact of strabismus on individuals. It has been reported that adults with strabismus complain of psychosocial problems; Satterfield et al. described the negative effect of strabismus on self-image, interpersonal relationships, and employment [2]. One study, strabismus was found to have a negative impact on self-image in about 70% of adults [3]. Olitsky et al. found that people with strabismus were more likely to be perceived as less intelligent, less competent, and having poorer communication skills than nonaffected people [4]. Paysse et al. reported that negative attitudes toward strabismic patients may develop around the age of six years [5]. Another study has shown that children with strabismus are more likely to develop mental illness by the third decade of life than those without strabismus [6].

Strabismus surgery aims to restore binocular vision, increase binocular fields, correct diplopia, or establish the normal alignment of the eyes [4]. At many times, if the patient is not diplopic or lacks the potential for binocular vision, strabismus surgery has often been labeled as purely cosmetic; however, this belief is incorrect. First, strabismus surgery is performed to correct an abnormality caused by a congenital or acquired defect in binocular vision [7]. Second, the presence of strabismus has been considered a disability because it has serious social consequences like the loss of normal eye contact, which may affect employment opportunities and interpersonal relationships [4].

Kushner et al. found that binocularity can be achieved in most patients undergoing strabismus surgery regardless of the type of preoperative deviation, duration of strabismus, or depth of amblyopia in the deviating eye (if present) [8]. Morris et al. also reported that adult patients with strabismus who have not been surgically aligned during early childhood might develop fusion after strabismus surgery [9]. Paysse et al. recommended early surgical correction for all types of strabismus to avoid the psychosocial stigma associated with this condition [5]. Hatt et al. found that health-related quality of life continues to improve from 6 weeks to 1 year following successful strabismus surgery, in both psychosocial and function domains, confirming the lasting benefits of strabismus surgery in such populations [10]. Menon and associates reported that 80% of patients with strabismus felt they had problems in their social lives and 95% of them noticed improved self-esteem and self-confidence after surgery [11]. Surgical correction of strabismus in adults was found to be safe and effective in improving ocular alignment, with success rates up to 85% [12].

Although the benefits of earlier surgical correction have been reported in the literature [8, 9, 13–15], it is common in our practice to see many adults with longstanding strabismus (more than one-year duration) who finally decided to have the surgery. Delay between the onset of strabismus and surgical correction is attributed to many factors, like common misconception about the complications and success rates of surgical intervention, nonaffordability, and lack of awareness among patients and some health care providers.

Many instruments have been developed to evaluate the health-related quality of life (HRQOL) among patients with strabismus. The Adult Strabismus Quality of Life Questionnaire (AS-20) is a strabismus specific one that assesses strabismus's psychosocial and functional impacts on affected individuals [16]. AS-20 showed high reliability and validity and was applicable to the majority of patients irrespective of their culture and economic status [17]. Chinese, Danish, and Italian versions of AS-20 showed acceptable validity and reliability for measuring (HRQOL) in patients with strabismus [18–20].

In this paper, we aim to find the reasons for delayed surgical correction and study the psychosocial and functional impacts of strabismus on adult population in Jordan using the AS-20 questionnaire. This is the first study conducted to address this issue in Jordan and the Middle East to the best of our knowledge.

## 2. Patients and Methods

This study included 79 patients with strabismus who had attended the outpatient ophthalmology clinics at two hospitals (Yarmouk Teaching Hospital and King Abdullah University Hospital) in Irbid, Jordan, between February 2021 and December 2021. The patients aged ≥18 years had strabismus for at least one year before surgical correction.

All patients underwent a comprehensive ophthalmic examination during their preoperative visit. A validated questionnaire was used to collect data on age, gender, social class, level of education, family history of strabismus in first degree relatives, onset and duration of strabismus, and the presence of diplopia. Social classes were determined based on average annual expenditure per household and divided into poverty, lower-middle, middle, upper-middle, and affluent classes as classified by the Jordanian Economic and Social Council [21]. Level of education was determined based on the highest educational qualification granted, and patients were classified accordingly into undergraduate (high school or below) or graduate (college or higher studies).

Patients were asked to select the most crucial reason behind the delay of surgical correction, and the following options were given:

(1). The ophthalmologist did not offer surgery.
(2). Surgery was offered but declined due to fear of surgical complications.
(3). Surgery was offered but declined because the patient thought he/she was too old for surgery or the patient was not bothered by the appearance.
(4). Surgery was offered but declined due to nonaffordability.
(5). The patient never sought care.

Moreover, the patients were asked to specify the reason to have surgical correction now. The following options were given: pressure/advice from family or friends, for cosmesis, to enhance chances of getting a job, for marriage/relationship prospects, having a better understanding of strabismus surgery and its potential complications, and improved economic status/medical insurance granted.

The AS-20 is a patient-derived quality of life questionnaire developed for adult strabismic patients [16]. The questionnaire consists of two subscales to measure strabismus's psychosocial (*P*) and functional (*F*) aspects. Each subscale consists of 10 questions and each question uses a 5-point Likert scoring system: “always” (score 0); “often” (score 25); “sometimes” (score 50); “rarely” (score 75); and “never” (score 100). For each subject, we calculated a mean overall score (i.e., the mean of the 20 items) and the mean scores for psychosocial and functional subscales (the mean of 10 items for each subscale), with a best-possible score of 100 (most favorable HRQoL) and worst, 0 (least favorable HRQoL). In the original AS-20 developed by Hatt et al., the threshold score for a normal, nonstrabismic subject was 84 [16].

In the current study, the questionnaire was translated from English to Arabic according to the World Health Organization (WHO) translation protocol. The primary author and a professor of English literature translated the English AS-20 into Arabic language. The synthetic Arabic version was then translated into English by two professional English translators who were not aware of the AS-20 questionnaire and were masked for the first translation. The back-translated copy of AS-20 was compared with the original version to look for any differences and then revised by a committee of health professionals (ophthalmologist, dermatologist, and epidemiologist). Then, a consensus was reached to develop a preliminary Arabic translation of the AS-20 questionnaire. The preliminary translation was then introduced to twelve strabismic patients to look for any confusion or ambiguous questions to the patients. All suggestions were taken into consideration to develop the final Arabic translation of the AS-20 questionnaire.

To address the cultural differences between our Arabic Middle-Eastern population and the USA population where the original questionnaire was run, a control group of 40 adults aged 18–65 without known visual defects was also recruited. Control subjects were patient companions, students, and health care providers. The translated AS-20 questionnaire together with the previous questions about sociodemographic data, reasons behind surgery delay, and the motivations for seeking treatment were administered to the 79 patients. In contrast, only the translated AS-20 questionnaire was administered to the 40 normal controls. The age and gender of controls were also recorded. All responses from patients and controls were reported and analyzed. All subjects answered the questions in the waiting room. Simple verbal and written instructions were provided to participants.

Our exclusion criteria for this study were patients under the age of 18, patients with strabismus of less than one-year duration, those not willing to participate in the study, lack of capacity to get verbal informed consent, and patients who had other ocular or facial abnormalities, such as facial nerve palsy or Graves' disease.

The research was performed in compliance with the tenets of the Declaration of Helsinki. The research protocol was approved by the Institutional Review Board of Yarmouk University. An informed consent was obtained from all patients and controls before enrolment.

## 3. Results

### 3.1. Participants' Characteristics

A total of 79 adult patients with strabismus (cases) and 40 subjects with normal vision (control group) were included in this study. The mean age (SD) was 34.10 (11.5) years for cases (range: 18–61) and 34.20 (11.2) years for controls (range: 18–65) (*p*=0.964). About half (55.7%, *n* = 44) of patients were males compared to (57.5%, *n* = 23) of controls. The sociodemographic characteristics of the patients are shown in Table 1. Almost two-thirds (64.6%) of patients had strabismus for ≥16 years.

### 3.2. Reasons for Strabismus Surgery Delay

The reasons for strabismus surgery delay and the motivations to undergo surgery now as reported by the patients are shown in Table 2. Not offering surgery by an ophthalmologist was the most frequently reported reason (35.4%) for the delay in surgical correction followed by fear from surgery complications (22.8%). The most common reasons to undergo surgery after this delay were to improve cosmetic appearance (27.8%) and developing a better understanding of strabismus surgery and its potential complications (20.3%).

### 3.3. The Psychosocial and Functional Impacts of Strabismus Using the AS-20 Questionnaire

The majority of patients (92.4%) believed that having strabismus was associated with psychological implications, and about 90% of them reported that they could have done better in their life without strabismus. When compared to controls, patients had significantly lower mean scores of total AS-20 (50.57 vs. 88.01) and psychosocial (49.59 vs. 87.84) and functional (51.55 vs. 88.19) subscales. Similarly, the means of AS-20 and its two subscales were significantly lower among patients compared to controls for men and women (Table 3).

In the multivariate analysis, females had significantly lower psychosocial, functional, and AS-20 mean scores than males by 8.78, 5.80, and 7.29 units, respectively. Patients with diplopia had higher mean psychosocial scores and lower mean functional scores than patients without diplopia. Increased age at onset by 1 year was associated with increased psychosocial score by 0.30. Increased size of deviation was significantly associated with decreased mean of psychosocial, functional, and AS-20 scores (Table 4).

**Figure 1:**
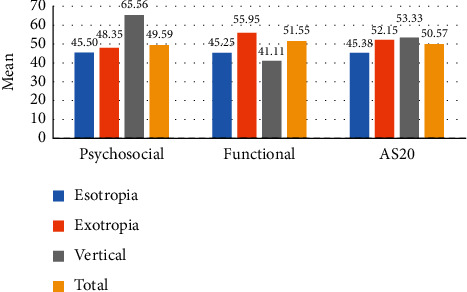
Types of deviation' impact on total AS-20, psychosocial, and functional mean scores.

Esotropia was associated with a significantly higher mean functional score (*B* = 8.66) compared to vertical deviation. Exotropia was also associated with a significantly higher mean functional score (*B* = 17.70) than vertical deviation. Esotropia had more negative impact on psychosocial, functional, and total AS-20 scores than exotropia as shown in Figure 1.

## 4. Discussion

Delaying surgical correction is common among our adult population with strabismus. The mean delay of surgical intervention was significantly higher among nondiplopic patients at 27.2 years than diplopic counterparts at 12.7 years. This is expected because diplopia has a significant functional impact on affected patients forcing them to seek surgical correction earlier than their nondiplopic counterparts, which was also demonstrated in previous studies [1, 22].

The current study showed that more than one-third of patients with strabismus surgery delay (35.4%) had not been offered an intervention by their health care provider who advised them that nothing could be done, adopted nonsurgical measures like prisms or patching of one eye for long period of time, or exaggerated the possible complications of surgical intervention; for example, we found that many health care providers and some ophthalmologists had exaggerated the risk of having persistent diplopia after surgical correction of longstanding strabismus pushing many patients to avoid surgery despite the small risk of developing this complication as reported by Kushner et al. [23]. This was also the most frequent reason for surgery delay in the study conducted by Coats et al. accounting for about 27% of the cases [1]. To address this issue, we have to educate health care providers about the importance of advising surgery in the appropriate time to save patients' unnecessary waiting times and the psychosocial and functional problems caused by their eye condition.

Another important reason for strabismus surgery delay was fear from surgery itself, accounting for (22.8%) of all cases. Some of these fears were exaggerated by a prior bad surgical experience of the patient or one of his/her relatives and friends, or due to common misconceptions about the complications of surgical intervention. This can be avoided by comprehensive preoperative counseling and addressing all patient concerns.

Nonaffordability came third on the list with about (18%) of the cases. Unfortunately, many patients did not have medical insurance; others did not have access to a specialized eye care, costing them years of suffering from a treatable eye condition. Government-offered and private health insurance policies should be reviewed to address this, among other health care issues. For example, strabismus surgery should be included for reimbursement and considered as a disease treatment rather than an optional cosmetic procedure.

Other delays were related to decreased patient awareness and could have been prevented by better public education, targeting patients who either thought they were too old for surgery or never sought care.

The present study also highlights the reasons behind having surgery after this long delay. More than a quarter of patients reported cosmesis as their primary motivation signaling the impact of cultural changes and the importance of enhancing physical appearance to improve chances of getting a job or developing successful relationships. This was also the main motivation for seeking surgery in a study conducted by Paduca et al. on Moldovan population [24].

About one-fifth of the patients decided to have surgery after developing a better understanding of strabismus surgery and its potential complications which can be attributed to improved patient-physician relationship and increased public awareness. Additionally, about 14% of the patients reported improved economic status as their primary reason to have surgery stressing the need to expand health care coverage and establishing specialized eye centers especially in rural areas.

The majority (92.4%) of surveyed patients admitted that strabismus had a negative psychological impact on them which has been reported in many other studies conducted on different populations [2, 4, 10, 11]. In this study, we used an Arabic version of the AS-20 HRQOL questionnaire to study strabismus' impacts on our patients. It revealed significantly lower scores of AS-20 and its two subscales among preoperative patients compared to control group; this is consistent with many other studies that used this instrument to measure the psychosocial and functional impacts of strabismus on adult patients [10, 16, 18–20, 25].

Patients in the present study seemed to have lower AS-20 scores than patients in other studies like the one conducted by Hatt et al. [10] with a preoperative median score of 59, which is significantly higher than our score of 50.57. On the other hand, our patients scored higher than those in the cohort conducted by Glasman et al. [25] at 45. This can be attributed to cultural and sociodemographic differences between the participants in those studies and ours.

In this study, patients with esodeviation had lower total AS-20, psychosocial, and functional scores than those with exodeviation, which corresponds well to previous studies that reported similar findings [4, 26, 27]. Nevertheless, in other studies, no difference was reported between the difficulties faced by those with exo-and esodeviations [2, 11, 28]. Patients with vertical deviations had more functional difficulties than those with horizontal deviations. In our sample, more patients with vertical deviations had diplopia than those with eso- and exodeviations which may explain their lower functional scores.

Female gender had a significant correlation with a lower AS-20, psychosocial, and functional scores. Although strabismus has a profound effect on facial expressions and eye contact in men and women, females are more likely to be prejudged according to their appearance than males, especially in Arabic communities, which may explain their lower psychosocial score. This was also reported by other studies which found that people rated females with strabismus more negatively than their male counterparts [27, 29, 30].

One of the limitations of this study is selection bias. Although this hospital-based study can provide us with a good idea about strabismus surgery delay and the impacts of strabismus on our patients, a population-based study may reveal more accurate numbers as many patients with strabismus do not seek specialized care. Those patients may not be adversely affected by strabismus in the same way the enrolled patients are; therefore, they may score themselves higher in HRQOL questionnaires.

Another limitation is using the original form of the AS-20 questionnaire rather than the modified Rasch-analyzed version recommended by the authors of the original AS-20. Although using the modified AS-20 may slightly change the numbers obtained in the present study, we do not feel that it would have a significant impact on them.

In conclusion, strabismus has profound psychosocial and functional impact on affected individuals, especially females and patients with large deviation sizes. Many adults with strabismus tend to delay surgical correction; most of these delays could be avoided by better public education, increased awareness among health care providers, and changing health insurance policies to cover the costs of strabismus surgery.

## Figures and Tables

**Table 1:** Sociodemographic and clinical characteristics of the patients (*n* = 79).

	*N*	%
Gender		
Female	35	44.3
Male	44	55.7
Age (years)		
18-29	30	38.0
30-44	31	39.2
≥45	18	22.8
Education		
Undergraduate	41	51.9
Graduate	38	48.1
Social class		
Poor	8	10.1
Lower-middle	38	48.1
Middle	25	31.7
Upper-middle	8	10.1
Affluent	0	0.0
Family history of strabismus		
No	54	68.4
Yes	25	31.6
Deviation type		
Esotropia	20	25.3
Exotropia	50	63.3
Vertical	9	11.4
Diplopia?		
No	54	68.4
Yes	25	31.6
Duration of strabismus (years)		
1–5	8	10.1
6–15	20	25.3
≥16	51	64.6
Age at diagnosis (years)		
<10	52	65.8
≥10	27	34.2
Size of deviation (PD)		
≤25	26	32.9
>25	53	67.1

**Table 2:** Reasons for strabismus surgery delay and seeking treatment now (n = 79).

*Reasons for surgery delay*	*N* (%)
Surgery was not offered by an ophthalmologist	28 (35.4)
Surgery was offered but declined due to fear from surgical complications	18 (22.8)
Surgery was offered but declined due to nonaffordability	14 (17.7)
Surgery was offered but declined because the patient thought he/she was too old for surgery or the patient was not bothered by the appearance	12 (15.2)
The patient never sought care	7 (8.9)

*Reasons for deciding to undergo surgery now*
For cosmesis	22 (27.8)
A better understanding of strabismus surgery and its potential complications	16 (20.3)
Pressure/advice from family or friends	13 (16.5)
Improved economic status/medical insurance granted	11 (13.9)
For marriage/relationship prospects	11 (13.9)
To enhance chances of getting a job	6 (7.6)

**Table 3:** The multivariate analysis of the association of the participants' sociodemographic and clinical characteristics with the total AS-20, psychosocial, and functional scores.

	Female				Male				Total			
	Controls		Cases		Controls		Cases		Controls		Cases	
	Mean	SD	Mean	SD	Mean	SD	Mean	SD	Mean	SD	Mean	SD
Psychosocial subscale	86.32	12.99	45.57	16.97	88.96	10.18	52.78	15.67	87.84	11.38	49.59	16.55
Functional subscale	86.62	10.68	46.71	12.36	89.35	7.73	55.40	11.98	88.19	9.08	51.55	12.83
Total AS-20	86.47	11.02	46.14	11.01	89.15	7.01	54.09	11.63	88.01	8.91	50.57	11.97

**Table 4:** Multivariate analysis of factors associated with total AS-20, psychosocial, and function mean scores among patients.

	Psychosocial				Function				Adult strabismus-20			
	*B*	95% Confidence interval		*p*-value	*B*	95% Confidence interval		*p*-value	*B*	95% Confidence interval		*p* value
Gender (female vs. male)	−8.78	−14.27	−3.30	0.002	−5.80	−10.60	−0.99	0.019	−7.29	−11.87	−2.71	0.002
Social class (poor-low vs. middle-affluent)	4.53	−1.18	10.24	0.118	1.16	−3.83	6.16	0.644	2.85	−1.91	7.61	0.237
Education (undergraduate vs. graduate)	3.27	−2.58	9.12	0.269	1.04	−4.08	6.16	0.687	2.15	−2.72	7.03	0.381
Deviation (esotropia vs. vertical deviation)	−1.77	−11.07	7.53	0.706	8.66	0.52	16.80	0.037	3.45	−4.31	11.20	0.379
Deviation (exotropia vs. vertical deviation)	8.39	−1.04	17.82	0.080	17.70	9.45	25.96	0.000	13.05	5.18	20.91	0.001
Diplopia (no vs. yes)	−5.80	−12.70	1.09	0.097	11.57	5.54	17.60	0.000	2.88	−2.86	8.63	0.320
Age at onset (<10 vs. ≥10 years)	0.30	0.02	0.58	0.034	0.13	−0.11	0.37	0.279	0.22	−0.01	0.45	0.066
Duration of strabismus	0.07	−0.16	0.31	0.528	0.14	−0.07	0.34	0.193	0.10	−0.09	0.30	0.288
Size of deviation (>25 vs. ≤25 PD)	−1.06	−1.30	−0.81	0.000	−0.41	−0.62	−0.20	0.000	−0.73	−0.94	−0.53	0.000

## Data Availability

The data used to support the findings of this study are available from the corresponding author upon request.

## References

[1] Coats D. K., Stager D. R., Beauchamp G. R. (2005). Reasons for delay of surgical intervention in adult strabismus. *Archives of Ophthalmology*.

[2] Satterfield D., Keltner J. L., Morrison T. L. (1993). Psychosocial aspects of strabismus study. *Archives of Ophthalmology*.

[3] Beauchamp G. R., Black B. C., Coats D. K. (2005). The management of strabismus in adults-III. The effects on disability. *Journal of American Association for Pediatric Ophthalmology and Strabismus*.

[4] Olitsky S. E., Sudesh S., Graziano A., Hamblen J., Brooks S. E., Shaha S. H. (1999). The negative psychosocial impact of strabismus in adults. *Journal of American Association for Pediatric Ophthalmology and Strabismus*.

[5] Paysse E. A., Steele E. A., McCreery K. M. B., Wilhelmus K. R., Coats D. K. (2001). Age of the emergence of negative attitudes toward strabismus. *Journal of American Association for Pediatric Ophthalmology and Strabismus*.

[6] McKenzie J. A., Capo J. A., Nusz K. J., Diehl N. N., Mohney B. G. (2009). Prevalence and sex differences of psychiatric disorders in young adults who had intermittent exotropia as children. *Archives of Ophthalmology*.

[7] Hunter D. G. (1995). Benefits of strabismus surgery in patients with one blind eye. *Archives of Ophthalmology*.

[8] Kushner B. J., Morton G. V. (1992). Postoperative binocularity in adults with longstanding strabismus. *Ophthalmology*.

[9] Morris R. J., Scott W. E., Dickey C. F. (1993). Fusion after surgical alignment of longstanding strabismus in adults. *Ophthalmology*.

[10] Hatt S. R., Leske D. A., Liebermann L., Holmes J. M. (2012). Changes in health-related quality of life 1 year following strabismus surgery. *American Journal of Ophthalmology*.

[11] Menon V., Saha J., Tandon R., Mehta M., Khokhar S. (2002). Study of the psychosocial aspects of strabismus. *Journal of Pediatric Ophthalmology & Strabismus*.

[12] Mills M. D., Coats D. K., Donahue S. P., Wheeler D. T. (2004). Strabismus surgery for adults: a report by the American academy of ophthalmology. *Ophthalmology*.

[13] Scott W. E., Kutschke P. J., Lee W. R. (1995). 20th annual Frank Costenbader lecture-adult strabismus. *Journal of Pediatric Ophthalmology & Strabismus*.

[14] Hertle R. W. (1998). Clinical characteristics of surgically treated adult strabismus. *Journal of Pediatric Ophthalmology & Strabismus*.

[15] Wortham E. V., Greenwald M. J. (1989). Expanded binocular peripheral visual fields following surgery for esotropia. *Journal of Pediatric Ophthalmology & Strabismus*.

[16] Hatt S. R., Leske D. A., Bradley E. A., Cole S. R., Holmes J. M. (2009). Development of a quality-of-life questionnaire for adults with strabismus. *Ophthalmology*.

[17] Leske D. A., Hatt S. R., Holmes J. M. (2010). Test–retest reliability of health-related quality-of-life questionnaires in adults with strabismus. *American Journal of Ophthalmology*.

[18] Yu H., Yang X., Ye T., Chen J., Zhang F., Yu X. (2013). Development and evaluation of a Chinese version of the adult strabismus questionnaire (AS-20). *Ophthalmic Epidemiology*.

[19] Ali N., Sørensen M. S., Sørensen T. L., Sørensen M. S., Mortzos P. (2016). Evaluation and validity of the Danish version of the adult strabismus questionnaire AS-20. *Clinical Ophthalmology*.

[20] Marcon G. B., Pittino R. (2014). The Italian version of the amblyopia and strabismus questionnaire: translation, validation, and reliability. *Strabismus*.

[21] Ababsa M. (2014). The socio-economic composition of the population. https://books.openedition.org/ifpo/5038.

[22] McBain H. B., Au C. K., Hancox J. (2014). The impact of strabismus on quality of life in adults with and without diplopia: a systematic review. *Survey of Ophthalmology*.

[23] Kushner B. J. (2002). Intractable diplopia after strabismus surgery in adults. *Archives of Ophthalmology*.

[24] Paduca A., Arnaut O., Lundmark P. O., Bruenech J. R. (2021). Causes of concomitant strabismus surgery delay in teenagers and adults. *Strabismus*.

[25] Glasman P., Cheeseman R., Wong V., Young J., Durnian J. M. (2013). Improvement in patients’ quality-of-life following strabismus surgery: evaluation of postoperative outcomes using the adult strabismus 20 (AS-20) score. *Eye*.

[26] Uretmen O., Egrilmez S., Kose S., Pamukçu K., Akkin C., Palamar M. (2003). Negative social bias against children with strabismus. *Acta Ophthalmologica Scandinavica*.

[27] Goff M. J., Suhr A. W., Ward J. A., Croley J. K., O’Hara M. A. (2006). Effect of adult strabismus on ratings of official USA army photographs. *Journal of American Association for Pediatric Ophthalmology and Strabismus*.

[28] Nelson B. A., Gunton K. B., Lasker J. N., Nelson L. B., Drohan L. A. (2008). The psychosocial aspects of strabismus in teenagers and adults and the impact of surgical correction. *Journal of American Association for Pediatric Ophthalmology and Strabismus*.

[29] Coats D. K., Paysse E. A., Towler A. J., Dipboye R. L. (2000). Impact of large angle horizontal strabismus on ability to obtain employment. *Ophthalmology*.

[30] Mojon-Azzi S. M., Potnik W., Mojon D. S. (2008). Opinions of dating agents about strabismic subjects’ ability to find a partner. *British Journal of Ophthalmology*.

